# Supplementary material for: Comparison of Growth Curve Estimates of Infants in São Tomé Island, Africa, with the WHO Growth Standards: A Birth Cohort Study
Source: Int J Environ Res Public Health. 2019 May 14;16(10):1693. doi: 10.3390/ijerph16101693 (PMC6572562; doi:10.3390/ijerph16101693)
Supplement: Supplementary file 1 [file ijerph-16-01693-s001.pdf]

Supplementary Table 1. Summary of quantile residuals for final length-for-age for boys model (BCPE($x = age^{0.35}$, $df(\mu) = 7$, $df(\sigma) = 2$, $\nu = 1$, $\tau = 2$).

Summary statistics	Value
Mean	0.00009247923
Variance	1.000342
Skewness	-0.04530505
Kurtosis	2.970894
Filliben correlation coefficient[a]	0.999553

[a] The Filliben correlation coefficient (Filliben, 1975) is a test for normality, where a result of 1 means normality.

Supplementary Table 2. Q-Statistics for the final model for length-for-age for boys.

Age (month)	Group	N	z1	z2	z3	z4	[a]Agostino K^2
-0.05 to 0.65	Birth	168	-0.49	-0.21	1.16	0.65	1.77
0.65 to 1.05	1 mo	141	0.92	0.17	1.44	1.35	3.90
1.05 to 2.05	2 mo	175	-0.87	0.75	-0.60	-1.29	2.03
2.05 to 3.05	3 mo	155	0.56	-0.03	1.19	0.05	1.42
3.05 to 4.05	4 mo	147	-0.12	-1.13	0.31	0.28	0.18
4.05 to 5.05	5 mo	167	-0.12	-0.08	1.47	0.17	2.19
5.05 to 6.05	6 mo	149	-0.59	-0.14	-1.65	-0.42	2.89
6.05 to 7.05	7 mo	155	0.97	-0.16	0.09	-1.52	2.33
7.05 to 8.15	8 mo	145	0.23	0.39	-1.21	1.15	2.79
8.15 to 9.15	9 mo	140	-0.17	-0.20	-0.97	-1.48	3.12
9.15 to 10.85	10 mo	156	0.18	0.32	-1.15	0.27	1.41
10.85 to 11.95	11 mo	167	0.13	0.61	-0.99	-0.35	1.09
11.95 to 13.15	12 mo	148	-0.23	-0.47	0.05	-0.10	0.01
13.15 to 14.65	14 mo	144	0.32	0.35	0.17	-1.36	1.88
14.65 to 16.05	16 mo	155	-0.35	-0.21	-1.53	0.78	2.94
16.05 to 18.15	18 mo	168	0.12	0.55	0.10	-0.20	0.05
18.15 to 20.45	20 mo	141	-0.48	-1.57	-0.15	0.79	0.65
20.45 to 22.95	22 mo	153	0.01	0.84	-1.14	0.89	2.08
22.95 to 24.85	24 mo	154	0.08	0.30	-0.89	0.89	1.60
Overall Q stats		2928	4.12	6.56	19.38	14.94	34.33
Degrees of freedom			10.0	16.5	19.0	19.0	38.0
p-value			0.942	0.985	0.432	0.726	0.640

Note: p-values are >0.05, therefore the model fits well; absolute values of z1, z2, z3 or z4 larger than 2 indicate misfit of, mean, variance, skewness or kurtosis, respectively, in corresponding age class; [a] D'Agostino's K^2 test for normality (D'Agostino, 1990).

Supplementary references

- Filliben, J.J. The probability plot correlation coefficient test for normality. *Technometrics*, **1975**, 17, *111-117.*
- D'Agostino, R.B.; Belanger, A.; D'Agostino Jr, R.B. A suggestion for using powerful and informative tests of normality. *Am. Stat.* **1990**, 44, 316-321.

Supplementary Table 3a. Median, minimum and maximum for weight-for-age (kg) measured in each point of assessment.

Age (month)	Male			Female		
	Median	Minimum	Median	Minimum	Median	Minimum
0	3.51	2.46	5.32	3.34	2.36	4.52
1	4.40	2.96	5.74	4.12	2.66	5.70
2	5.52	3.86	7.19	5.18	3.17	6.71
3	6.35	4.72	8.15	5.86	3.85	8.16
4	7.02	5.30	8.80	6.41	3.98	8.61
5	7.38	5.44	9.40	6.91	4.80	9.15
6	7.83	5.46	10.40	7.34	5.14	9.45
7	8.17	5.28	10.44	7.50	5.63	9.90
8	8.31	6.78	11.48	7.70	5.60	10.54
9	8.45	6.56	12.11	7.96	5.47	10.74
10	8.87	6.53	12.36	8.22	5.65	10.80
11	8.96	6.42	12.44	8.24	6.16	10.91
12	9.22	7.37	12.74	8.58	6.20	11.40
13	8.87	7.14	10.44	8.46	6.33	10.63
14	9.68	7.71	13.37	9.19	6.73	11.99
15	9.19	8.12	11.09	8.93	7.11	11.15
16	10.07	8.11	13.55	9.66	7.15	12.34
17	10.10	8.81	11.67	9.26	7.41	11.62
18	10.48	8.31	14.08	10.02	7.69	13.78
19	10.80	9.23	11.81	10.23	8.37	12.60
20	10.75	8.33	13.73	10.36	8.03	13.86
21	10.80	9.04	13.31	10.38	8.36	12.22
22	11.05	8.46	14.78	10.54	7.98	14.14
23	11.39	9.25	13.20	10.82	8.66	12.97
24	11.59	9.20	15.71	11.14	8.39	14.71

Supplementary Table 3b. Median, minimum and maximum for length-for-age (cm) measured in each point of assessment.

Age (month)	Male			Female		
	Median	Minimum	Median	Minimum	Median	Minimum
0	50.80	45.00	56.20	49.70	45.30	54.60
1	53.40	49.20	59.60	52.45	48.00	57.80
2	57.20	52.60	64.50	55.95	51.00	61.00
3	60.80	55.70	66.10	59.00	55.00	63.80
4	63.25	58.50	68.20	61.20	55.00	66.00
5	64.80	60.30	70.60	63.20	59.20	68.00
6	66.70	60.90	73.00	65.00	60.70	70.80
7	68.70	61.20	73.20	66.40	62.60	71.80
8	70.00	65.20	75.50	67.90	62.00	72.80
9	71.30	65.00	75.60	69.50	62.80	75.30
10	72.60	66.30	78.30	70.70	64.50	76.20
11	74.00	67.30	79.80	71.40	66.50	77.40
12	74.90	69.00	81.50	72.70	65.40	78.50
13	74.60	70.50	80.40	73.00	67.50	78.80
14	77.00	70.70	83.00	74.70	68.30	80.90
15	77.00	70.50	82.40	75.30	69.50	81.70
16	79.00	72.80	85.60	77.10	70.30	83.30
17	78.60	74.60	83.00	76.90	73.20	81.30
18	80.35	73.20	87.20	79.00	72.70	87.00
19	81.50	76.50	84.30	80.35	75.50	83.40
20	82.65	74.40	89.90	81.30	74.40	88.80
21	83.20	77.00	90.30	81.40	76.70	86.80
22	84.70	75.70	92.30	83.15	76.50	91.40
23	84.20	80.30	90.70	83.30	77.70	87.70
24	86.50	77.50	95.40	84.70	77.80	94.20

Supplementary Table 3c. Median, minimum and maximum for weight-for-length (kg/cm) measured in each point of assessment.

Length (cm)	Male				Female	
	Median	Minimum	Median	Minimum	Median	Minimum
46	2.55	2.46	2.60	2.61	2.49	3.14
48	2.80	2.53	3.57	3.04	2.36	3.77
50	3.32	2.46	4.11	3.39	2.81	4.26
52	3.85	3.10	4.95	3.92	3.13	4.87
54	4.46	3.56	5.78	4.52	3.39	5.70
56	5.01	3.88	6.90	5.08	3.85	6.16
58	5.60	4.44	7.01	5.57	4.52	7.08
60	6.19	4.65	8.11	6.01	4.63	7.84
62	6.65	5.26	8.80	6.56	5.18	8.69
64	7.21	5.82	9.52	6.97	5.24	9.15
66	7.57	6.05	9.78	7.35	5.86	9.45
68	7.96	6.58	10.40	7.64	6.00	10.54
70	8.23	6.42	10.29	8.00	6.16	10.74
72	8.59	7.14	11.25	8.35	6.26	11.10
74	9.05	7.20	11.44	8.73	7.11	11.72
76	9.40	7.72	12.36	9.24	8.00	11.99
78	9.84	8.17	12.74	9.68	7.98	12.34
80	10.25	8.96	12.66	10.17	8.39	12.26
82	10.78	8.46	13.43	10.46	8.72	12.60
84	11.01	9.68	14.08	10.71	9.39	12.78
86	11.46	9.90	13.73	11.37	10.01	13.36
88	11.93	10.00	14.22	11.67	9.89	13.86
90	12.70	10.90	15.71	12.18	10.70	13.28
92	12.75	11.48	13.81	13.02	11.91	14.14
94	12.42	12.08	14.10	12.30	12.30	14.71

Supplementary Table 3d. Median, minimum and maximum for body mass index-for-age (kg/m^2) measured in each point of assessment.

Age (month)	Male			Female		
	Median	Minimum	Median	Minimum	Median	Minimum
0	13.50	10.08	18.31	13.55	10.55	16.72
1	15.10	12.37	19.47	15.00	11.31	18.28
2	16.63	13.18	21.31	16.26	12.19	19.86
3	17.33	14.00	21.28	16.67	12.45	21.50
4	17.46	14.32	22.84	16.96	12.47	21.69
5	17.51	14.42	23.41	17.19	13.25	22.31
6	17.65	14.27	23.03	17.20	13.12	21.80
7	17.30	13.49	22.87	17.06	13.75	22.05
8	17.02	14.13	22.19	16.77	13.63	22.33
9	16.70	13.92	21.47	16.53	13.05	22.11
10	16.81	13.06	21.40	16.33	13.39	21.42
11	16.59	13.33	20.87	16.18	12.57	20.76
12	16.45	13.50	21.27	16.10	12.28	20.94
13	16.10	13.66	18.94	15.76	13.53	18.73
14	16.35	13.77	20.13	16.23	13.37	21.52
15	16.01	13.29	19.45	15.84	13.19	19.17
16	16.23	13.92	19.89	16.17	12.92	20.13
17	16.47	14.72	19.56	15.64	13.35	19.25
18	16.18	13.33	19.95	16.11	13.19	19.12
19	16.43	14.92	18.39	15.73	14.11	18.47
20	15.76	13.48	19.58	15.67	13.36	18.04
21	15.60	13.91	18.43	15.35	13.29	17.68
22	15.60	12.80	18.62	15.26	13.02	18.09
23	15.57	14.03	18.69	15.67	13.23	18.26
24	15.49	13.03	19.48	15.28	12.71	18.45

Supplementary Table 3e. Median, minimum and maximum for head circumference-for-age (cm) measured in each point of assessment.

Age (month)	Male			Female		
	Median	Minimum	Maximum	Median	Minimum	Maximum
0	35.80	33.20	39.60	35.20	32.50	38.80
1	37.50	34.70	41.00	36.90	34.40	40.20
2	39.35	36.70	43.00	38.65	36.20	41.70
3	40.80	38.30	44.10	39.95	37.30	43.30
4	42.20	39.20	46.00	41.13	38.50	45.00
5	42.90	40.50	46.70	42.00	39.20	45.80
6	43.85	41.10	47.50	42.75	39.80	47.30
7	44.50	42.20	48.90	43.45	39.50	46.70
8	45.00	41.70	47.90	43.90	41.00	47.40
9	45.40	42.80	49.20	44.50	40.80	48.20
10	45.70	43.00	49.80	44.80	41.00	48.20
11	45.70	42.70	49.60	44.80	41.30	48.00
12	46.00	43.70	50.00	45.20	41.30	48.00
13	46.00	43.70	49.10	45.00	43.00	48.20
14	46.70	44.00	51.00	45.70	42.00	48.80
15	46.40	44.10	49.50	45.70	43.00	48.50
16	46.80	44.30	51.50	46.00	42.20	48.90
17	46.60	45.20	49.70	45.70	44.00	48.20
18	47.00	44.50	51.30	46.25	43.00	49.50
19	47.25	46.20	49.00	46.50	44.50	48.90
20	47.50	45.00	53.10	46.70	42.90	49.10
21	48.05	45.50	53.60	47.00	44.00	50.00
22	48.00	45.70	50.80	47.30	43.20	50.20
23	47.70	46.00	50.70	47.30	45.30	50.00
24	48.30	45.70	56.40	47.50	43.30	50.20

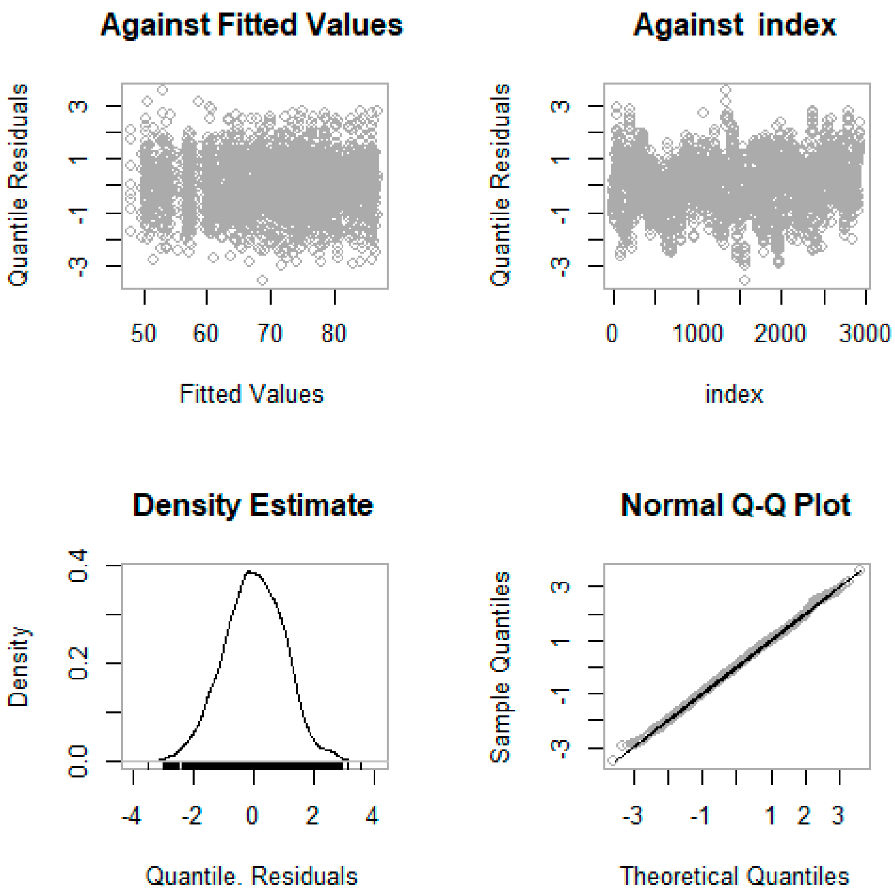

Supplementary Figure 1. Normality study of the residuals of the final model for length-for-age for boys.

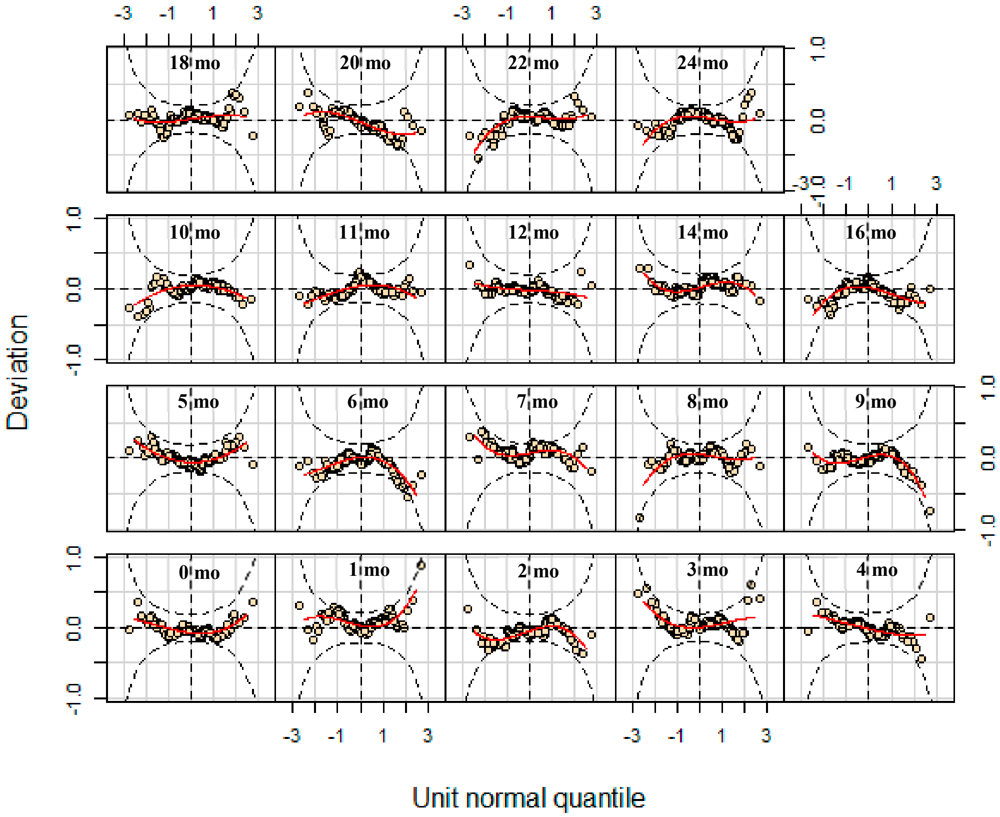

Supplementary Figure 2. Worm plots of the final model for length-for-age for boys. Each panel represents individuals in an age group (months). Dashed semicircles represent 95% confidence intervals. Most points fit within the 95% confidence intervals, thus the model fits well.

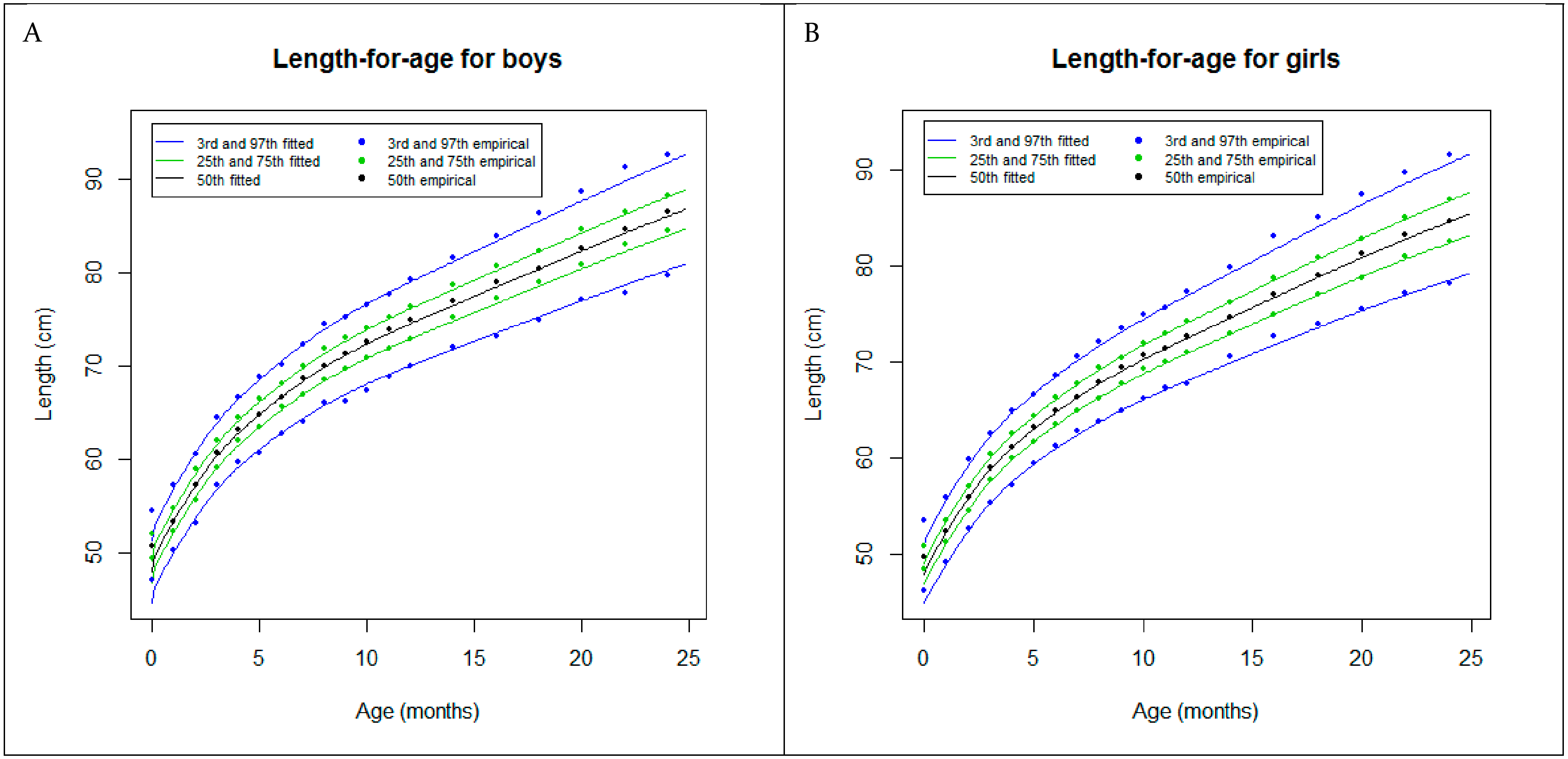

Supplementary Figure 3. Comparison of smoothed percentile curves (solid lines) and empirical values: length-for-age for boys (A) and girls (B) from birth to 24 months of age. Smoothed percentile curves overlapping empirical values mean a perfect fit of the model.
